# Influence of switching rule on motor learning

**DOI:** 10.1038/s41598-018-31825-4

**Published:** 2018-09-10

**Authors:** Kotaro Ishii, Takuji Hayashi, Ken Takiyama

**Affiliations:** Department of Electrical and Electronic Engineering, Tokyo University of Agriculture and Technology, Koganei-shi, Tokyo 184-8588 Japan

## Abstract

Humans and animals can flexibly switch rules to generate the appropriate response to the same sensory stimulus, e.g., we kick a soccer ball toward a friend on our team, but we kick the ball away from a friend who is traded to an opposing team. Most motor learning experiments have relied on a fixed rule; therefore, the effects of switching rules on motor learning are unclear. Here, we study the availability of motor learning effects when a rule in the training phase is different from a rule in the probe phase. Our results suggest that switching a rule causes partial rather than perfect availability. To understand the neural mechanisms inherent in our results, we verify that a computational model can explain our experimental results when each neural unit has different activities, but the total population activity is the same in the same planned movement with different rules. Thus, we conclude that switching rules causes modulations in individual neural activities under the same population activity, resulting in a partial transfer of learning effects for the same planned movements. Our results indicate that sports training and rehabilitation should include various situations even when the same motions are required.

## Introduction

While kicking a soccer ball toward a friend, recreational soccer players frequently fail to achieve the planned movement. After the ball deviates from the intended movement direction, the players modify their motor commands when performing their next action. Humans and animals have the ability of motor learning to decrease prediction errors between planned and actual movements^1–5^. The features of motor learning have been clarified based on visually guided and goal-directed arm-reaching movements. After learning reaching movements toward a movement direction, motor learning effects are partially available for reaching movements toward other movement directions^6,7^. Motor learning effects can differ when the executed actions are the same but the planned movement directions are different^8,9^. Based on the transfer of learning effects (a possible measure of the relation between two movement patterns), the relation among movement patterns depends on the differences in the planned movement directions.

Previous studies have relied on the following fixed rule: the same instructed target requires the same planned movements. However, humans and animals can act flexibly toward the same sensory stimulus, e.g., we kick a soccer ball toward a friend who belongs to our team, but we kick the ball away from a friend who is traded to an opposing team (here, we consider only “raw” sensory stimuli without considering any effects of context on the perceived sensory stimuli). This relation between the sensory stimulus and response is the stimulus-response map (S-R map)^10,11^. Humans and animals can flexibly switch between congruent (movement direction is toward a sensory stimulus, e.g., pro-saccade and pro-reaching movement) and incongruent (movement direction is away from a sensory stimulus, e.g., anti-saccade and anti-reaching movement) S-R maps. Thus, we can plan the same movements for different targets using various rules, e.g., kicking toward a rightward direction when a friend in our (opposing) team is in the rightward (leftward) direction. Because conventional motor learning experiments have relied on one rule (i.e., the congruent S-R map) and suggested that the planned movement direction determines the motor learning effects, whether the same motor learning effect applies when the planned movement is the same but the rule is switched is unclear.

Here, we study the effects of switching rules on motor learning using pro- and anti-arm-reaching movements and a visuomotor rotation task^12^. Based on previous behavioral and neurophysiological evidence, the following two possibilities are considered. First, the transfer of learning effects is 100% in the same planned movement direction when a rule is switched. This possibility is based on the following behavioral evidence: motor learning experiments that relied on the congruent S-R map^8,9^ and a previous study that investigated the transfer from pro- to antisaccades in saccadic gain adaptation^13,14^. Second, the transfer is not 100% when the rule is switched even when the planned movement direction is the same. This possibility is based on the following neural evidence: different neural activities have been observed between pro- and anti-reaching movements^15,16^ and between pro- and antisaccades^10^ in several brain regions. Certain conventional motor learning theories (e.g., the framework of motor primitives) are inspired by neural activities^6^ and the difference in neural activities may be responsible for the less than 100% transfer of learning effects between pro- and anti-reaching movements.

The current study supports the second possibility as follows. Switching rules cause partial availability of motor learning effects even in the same planned movement direction. Although motor learning effects can be decomposed into at least two components, explicit (cognitive) or implicit components, we investigated the implicit components following previous studies^3,17,18^. To investigate the effects of switching rules on learning effects in detail, we constructed a computational model. Our computational model indicates that each neural unit has a different activity, but the population activity is constant in the same planned movement with a different rule condition.

## Materials and Methods

### Participants

Forty healthy, right-handed volunteers (aged 18–26 years, nine women) participated in our experiments (10 participants in each of the four experiments). All participants were informed of the experimental procedures in accordance with the Declaration of Helsinki and all participants provided written informed consent prior to the initiation of the experiments. All procedures were approved by the ethics committee of the Tokyo University of Agriculture and Technology.

### Experimental Design and Statistical Analysis

The participants were asked to perform 8-cm arm-reaching movements with their right arm while holding the handle of a manipulandum (Geomagic 1.5 HF; Geomagic, Rock Hill, SC, USA). The position of the handle was displayed as a white cursor (6-mm circle) against a black background on a horizontal screen located above a participant’s hand. When the visuomotor rotation was introduced, the cursor position deviated from the handle position by multiplying a rotation matrix.

The movement of the handle was constrained to a virtual horizontal plane (10 cm below the screen) that was implemented by a simulated spring (1.0 kN m^−1^) and damper (0.1 N per (ms^−1^)). A brace was used to reduce unwanted wrist movements. Before each trial, the participants were required to hold the cursor at its center circle (a 10-mm circle). The position and velocity data of the handle were recorded at 500 Hz.

#### Experiment 1

**Figure 1:**
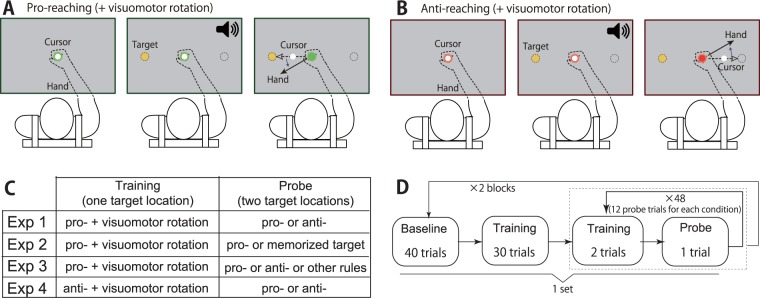
Schematic of the pro- and anti-reaching movements. (**A**) Participants performed arm-reaching movements to move the white cursor from the center circle to a target. When the color of the center circle was green, the participants were required to move the white cursor toward the yellow visual cue (pro-reaching). A beep sounded at the same time of the appearance of the yellow visual cue to signal the participant to initiate an arm-reaching movement. During the training trials, a visuomotor rotation was imposed. Visuomotor rotation caused the invisible hand movement direction (solid black line) to deviate from the visible cursor movement direction (dotted black line). (**B**) When the color of the center circle was red, the participants were required to perform anti-reaching movements to move the white cursor toward the opposite location of the yellow visual cue. (**C**) Experimental protocol for experiments 1, 2 and 4. In experiment 1, participants performed two sets of trials, which each consisted of 40 baseline trials (20 trials with an invisible cursor trajectory), 30 training trials with a visuomotor rotation and pro-reaching movements and 48 probe trials with an invisible cursor trajectory. Trials with an invisible cursor trajectory prevented online correction. Before each probe trial, two training trials with a visible cursor trajectory, visuomotor rotation and pro-reaching movements were performed in experiment 1. In experiment 4, pro-reaching movements in training trials were substituted for anti-reaching movements. In experiment 2, anti-reaching movements in baseline and probe trials were substituted for reaching movements for to-be-memorized targets. (**D**) Summary of each experiment.

After a 2-s holding time in the center circle (10-mm circle), the color of the center circle changed according to the experimental conditions. Green and red colors indicated pro- and anti-reaching trials, respectively. In the pro-reaching trials, the participants were required to perform arm-reaching movements to move the white cursor toward the visual cue (a 10-mm circle), the color of which was yellow (Fig. 1A). In the trials with the anti-reaching movements, the participants were required to perform arm-reaching movements to move the white cursor toward a position opposite to the visual cue (Fig. 1B). After an additional 1-s holding time, a yellow visual cue appeared at either the 180° or 0° locations (180° and 0° locations indicated the 9 o’clock and 3 o’clock directions, respectively). With the same timing as the appearance of the cue, a beep sounded to signal the participant to initiate an arm-reaching movement. This setting was similar to reactive rather than voluntary eye movements^13,14^. The participants were required to move the handle at a peak velocity of 470 ± 45 mm s^−1^ (the target velocity was calculated using the minimum-jerk theory with a movement amplitude of 8 cm and a duration of 0.4 s). A warning message appeared on the screen if the movement velocity of the handle rose above (“fast”) or fell below (“slow”) this threshold value. No online correction was allowed. At the end of each trial, the handle was automatically moved back to the starting position by the manipulandum. The participants practiced using the manipulandum and became accustomed to the experimental settings during 98 trials (70 trials for pro-reaching movements and 28 trials for anti-reaching movements).

A visuomotor rotation was applied in the pro-reaching movements and the transfer of learning effects from the pro- to the anti-reaching movements was investigated in experiment 1 (Fig. 1C). After the practice trials, participants experienced 2 blocks, which each comprised 40 baseline, 126 training and 48 probe trials. In baseline trials, subjects pseudorandomly performed pro- and anti-reaching movements toward either the 180° or 0° locations. In training trials, subjects performed pro-reaching movements toward a fixed target location (i.e., 180° or 0° locations but the location was the same across all training trials) with visuomotor rotations. The target location in training trials was counterbalanced across blocks and subjects. Because the manner in which visuomotor rotation is introduced could impact learning effects^12^, the participants experienced two different types of visuomotor rotation in each set as follows: a 15-degree visuomotor rotation that was abruptly introduced and a visuomotor rotation that increased by 0.5 degrees in each trial and reached a maximal value of 15 degrees. The order of the two types of visuomotor rotations (abruptly applied or gradually increasing), the direction of the visuomotor rotation (clockwise or counterclockwise) and the target direction in the training trials were counterbalanced across all blocks and participants. In probe trials, the current study probed learning effects by hiding the white cursor. Because learning effects obtained in pro-reaching movements were partially available in anti-reaching movements (see Results), the prediction error could be observed in anti-reaching movements and the error could affect the following pro- and anti-reaching movements. Thus, we hid the white cursor in probe trials to eliminate the effects of the sensory prediction error on anti-reaching movements in the following training and probe trials; however, hiding the cursor could induce a reduction in learning effects^19^. To prevent a reduction in learning effects in the next probe trials, we introduced two training trials after each probe trial. A set of two training trials with the white cursor displayed and one probe trial to calculate *θ*_probe,i_, with the white cursor hidden were repeated 48 times. A short break was provided after 20 repetitions (Fig. 1D). Because the short break caused the participants to forget the learning effects, ten learning trials with a 15-degree visuomotor rotation were added after the short break. After one set, 36 washout trials were introduced.

We calculated the learning effects as follows. In 20 of the 40 baseline trials, the white cursor was hidden to calculate the baseline movement direction *θ*_baseline,i_ (i.e., 5 baseline trials for each type of trial). The baseline movement directions were also calculated by hiding the white cursor before introducing the visuomotor rotation to fairly compare the movement directions in probe trials to those in baseline trials. The learning effect was calculated as ${\hat{\theta }}_{{\rm{probe}}}-{\hat{\theta }}_{{\rm{baseline}}}$, where ${\hat{\theta }}_{{\rm{probe}}}$ and ${\hat{\theta }}_{{\rm{baseline}}}$ indicated the movement direction (calculated based on the manipulandom position) after and before adapting to the visuomotor rotation, respectively. ${\hat{\theta }}_{{\rm{probe}}}$ and ${\hat{\theta }}_{{\rm{baseline}}}$ were calculated as ${\hat{\theta }}_{{\rm{probe}}}=\frac{1}{{T}_{{\rm{p}}}}{\sum }_{i=1}^{{T}_{{\rm{p}}}}{\theta }_{{\rm{probe}},{\rm{i}}}$ and ${\hat{\theta }}_{{\rm{baseline}}}=\frac{1}{{T}_{{\rm{b}}}}{\sum }_{i=1}^{{T}_{{\rm{b}}}}{\theta }_{{\rm{baseline}},{\rm{i}}}$, where *T*_p_ was the number of successful trials during the probe phase, *θ*_probe,i_ was the movement direction at the *i* th successful trial during the probe phase (calculated based on the manipulandom position), *T*_b_ was the number of successful trials during the baseline phase and *θ*_baseline,i_ was the movement direction at the *i* th successful trial during the baseline phase. Because the participants occasionally performed pro-reaching movements during the anti-reaching trials and anti-reaching movements during the pro-reaching movements, *θ*_probe,i_ and *θ*_baseline,i_ were calculated for the successful trials only. We determined the success of each trial according to the movement angle as follows: when the movement angle at all movement times was ±90 within the required movement direction, the trial was considered a success. The movement directions *θ*_probe,i_ and *θ*_baseline,i_ were calculated when the movement velocity reached its peak value.

Although recent studies have revealed that the learning effects in the visuomotor rotation task comprised at least two components, explicit and implicit learning effects^3,17,18^, our primary interest in the current study was not cognitive or explicit learning effects but implicit learning effects. To investigate the implicit learning effects, we instructed all participants to strictly aim at the visible target in pro-reaching movements and the position opposite the target in anti-reaching movements in any situation, consistent with previous studies^3,17,18^.

#### Experiment 2

In our experimental settings, the anti-reaching movements required arm-reaching movements toward a location without a visual cue (i.e., invisible target). To investigate the effect of reaching toward the invisible target, we conducted experiment 2. In experiment 2, a visuomotor rotation was applied to pro-reaching movements toward a visual cue and the transfer of the learning effects from those reaching movements to pro-reaching movements toward an invisible and a to-be-memorized target location was investigated (Figs 1C, 3A,B). After a 2-second holding time at the starting position, the color of the center circle changed according to the experimental condition and a yellow visual cue (a 10-mm circle) appeared at either the 0° or 180° location. When the center circle was red, the target disappeared after 400 ms. Green and red colors indicated reaching movements toward the visual cue and to-be-memorized targets, respectively. After an additional 1-s holding time, a beep sounded, signaling the participants to initiate an arm-reaching movement to move the white cursor toward the target. The participants practiced using the manipulandum and became accustomed to the experimental settings over 98 trials (70 trials for reaching movements toward visible targets and 28 trials for reaching movements toward invisible and to-be-memorized targets).

During the baseline, training and probe trials, the target location and whether the target was to be visible or to-be-memorized were determined following a process similar to that performed in experiment 1, except for that the anti-reaching movements in experiment 1 were substituted for pro-reaching movement toward invisible and to-be-memorized targets in experiment 2.

#### Experiment 3

To determine whether the anti-reaching movements followed a particular type of rule switching that caused partial availability of the learning effects, we conducted experiment 3. In experiment 3, a visuomotor rotation was applied to pro-reaching movements and the transfer of the learning effects from the pro-reaching movements to other reaching movements using the switching rule was investigated (Fig. 1C). After a 2-second holding time at the center circle (a 10-mm circle), a green or red visual cue appeared at either the 0°, 90°, 180°, or 270° location (in total, there were eight types of targets). With the same timing as the cue appearance, a beep sounded, signaling the participants to initiate the arm-reaching movement toward the target (i.e., the visual cue in pro-reaching movements and the position opposite the cue in anti-reaching movements). The participants were required to perform 8-cm arm-reaching movements to move the white cursor toward the 180° location when the target color was green (Fig. 4A) and toward the 0° location when the color was red independently of the cue location (Fig. 4B). One second after the beep sounded, a gray circle appeared at the location where the participants were required to aim (i.e., a gray circle appeared in the leftward location when the target color was green). The participants practiced using the manipulandum and became accustomed to the experimental settings over 118 trials (78 trials with a yellow target in which the participants were required to perform arm-reaching movements toward the yellow cue and 40 trials in which the participants performed reaching movements toward either the 180° or 0° location depending on the color of the visual cue [green for 180° and red for 0°] that appeared at either 0°, 90°, 180°, or 270°).

All participants performed a set of trials comprising 80 baseline (target color [either green or red] and target location [either 0°, 90°, 180°, or 270°] were pseudorandomly determined), 236 learning (target color and location were fixed to either green and 180° or red and 0° for each subject, i.e., pro-reaching movements; Fig. 4A) and 96 probe (target color and location were pseudorandomly determined) trials. In 40 of the 80 baseline trials, a white cursor was hidden to calculate the baseline movement direction *θ*_baseline,i_ (i.e., 5 baseline trials for each type of trial). In the following 44 learning trials, a visuomotor rotation was imposed. The visuomotor rotation increased by 0.5 degrees in each trial and reached a maximal value of 15 degrees. In the remaining trials, a block of two training trials with the white cursor displayed and one probe trial to calculate *θ*_probe,i_ with the white cursor hidden were repeated 96 times.

Two short breaks were provided after 32 blocks. Because the short break caused the participants to forget the learning effects, ten learning trials with a 15-degree visuomotor rotation were added after the short break. In the learning trials, the visuomotor rotation was increased by 0.5 degrees in each trial and reached a maximal value of 15 degrees. The direction of the visuomotor rotation, the target color and the location in the learning and training trials were counterbalanced across all participants.

#### Experiment 4

We conducted experiment 4 to determine whether the partial availability of the learning effects was specific to the probing of the transfer from the pro- to the anti-reaching movements. In experiment 4, a visuomotor rotation was applied to the anti-reaching movements and the transfer of the learning effects from the anti- to the pro-reaching movements was investigated (Fig. 1C). The experimental settings and protocols were similar to those in experiment 1. However, the training trials were performed using anti-reaching movements. One second after a beep sounded, a gray circle appeared at the location where the participants were required to aim (e.g., a gray circle appeared at the leftward location when the visual cue was rightward in the anti-reaching movements). The participants practiced using the manipulandum and became accustomed to the experimental settings over 98 trials (70 trials for the pro-reaching movements and 28 trials for the anti-reaching movements).

During the baseline, training and probe trials, the target location and the pro- or anti-reaching movements were determined using a similar process to that used in experiment 1, except for that the pro-reaching movements in the training trials in experiment 1 were substituted for anti-reaching movements in experiment 4.

#### Statistical Analysis

Repeated measures analyses of variance (ANOVAs) were conducted if there were no specifications regarding statistical tests. If significant interactions among factors occurred, ANOVA was followed by Tukey’s post hoc comparisons. The current study considered three within-subject factors in experiments 1, 2 and 4, including ‘Type of reaching movements’ (pro- or anti-reaching movements in experiments 1 and 4, reaching movements toward a visible or to-be-memorized target in experiment 2), ‘Movement direction’ (trained or nontrained movement directions) and ‘Type of perturbation’ (abruptly introduced or gradually increased). In experiment 3, repeated measures ANOVAs were conducted with consideration of the following factors: ‘Type of reaching movements’ (pro-reaching movements, reaching movements toward ±90 rotated location, or anti-reaching movements) and ‘Movement direction’ (trained or nontrained movement directions). All statistical analyses were performed using MATLAB (Mathworks Inc.).

### Computational model

We relied on the motor primitive framework^4,6,7,20^. In this framework, an additional motor command, *x*_*t*_ at the *t* th trial, was modeled as a linear combination of neural activities *A*_*i*_ (*i* = 1, ..., *N*, where *N* was the number of neural units); ${x}_{t}={\sum }_{i=1}^{N}\,{W}_{i,t}{A}_{i}={{\boldsymbol{W}}}_{t}{{\bf{A}}}_{t}$, where ***W***_*t*_ = (*W*_1,*t*_, ..., *W*_*N*,*t*_) and ***A***_*t*_ = (*A*_1,*t*_, ..., *A*_*N*,*t*_)^*T*^. For simplicity, we focused on a single movement direction. First, we calculated the transfer of the learning effects between the pro- and anti-reaching movements in the case of the general functional form of ***A***_*t*_. Next, we assumed a simple form of ***A***_*t*_. In the current study, we considered the case when *A*_*i*,*t*_ = *A*_*i*_ + *ξ*_*i*,*t*_, where *ξ*_*i*,*t*_ was trial-to-trial variant noise with a mean, standard deviation and covariance of 0, *σ*^2^ and Cov(*ξ*_*i*,*t*_, *ξ*_*j*,*t*_) = *σ*^2^*δ*_*ij*_, respectively (*δ*_*ij*_ = 1 when *i* = *j* and 0 otherwise), such as fluctuations in the aiming direction. Although the current study focused on this simplified version of noise, the following calculations could be applicable to the general case of *ξ*. Our program codes are available in the Supplementary material and on our website.

#### Transfer of learning effects

**Figure 2:**
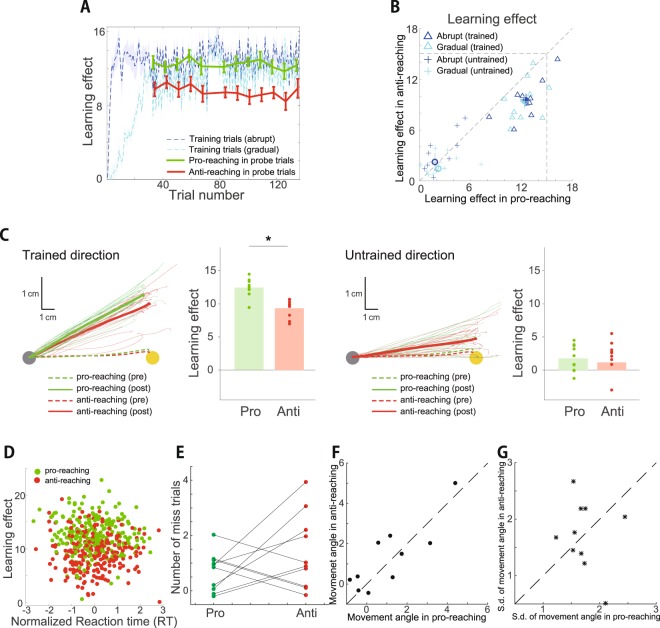
Transfer of learning effects from pro- to anti-reaching movements. (**A**) Learning curves during the training (dotted blue and cyan lines in the former 126 trials) and probe trials (solid green and red lines for pro- and anti-reaching movements in the probe trials, respectively). Horizontal and vertical axes denote the trial number and learning effect, respectively (the definition of learning effect is provided in the *Materials and Methods* section). Blue and cyan lines denote the learning curves for the visuomotor rotation that was abruptly introduced and gradually increased, respectively. Blue and cyan shaded area denote the standard error of the mean (s.e.m.) (N = 10). Green and red error bars also denote the s.e.m. (N = 10). (**B**) Learning effects in each participant. Open triangles and filled circles denote the learning effects in each participant and mean learning effects in reaching for the trained movement direction. Solid lines crossing the filled circles denote the s.e.m. (N = 10). Blue and cyan colors indicate learning effects when the visuomotor rotation was introduced abruptly and gradually increased, respectively. Blue and cyan crosses denote learning effects in reaching for the non-trained movement direction. Open circles and solid lines crossing the circles denote the mean and s.e.m. of the learning effects. (**C**) Trajectories of reaching movements and learning effects in pro- (green) and anti-reaching (red) movements during the probe trials. All trajectories were arranged to be directed toward an up-rightward direction. (Left): Trajectories toward the trained movement direction. Thin lines indicate the mean trajectory of each participant during the probe trials in each set. The thick solid lines indicate the mean trajectories across all participants and all sets. Thick dotted lines indicate the mean trajectories in all training trials. (Center left): Averaged learning effects in the trained movement direction across all sets. Each dot denotes the learning effects in each block. Asterisk indicates a significant difference (Turkey’s post hoc test). (Center right): Trajectories toward the untrained movement direction. (Right): Learning effects in the untrained movement direction. (**D**) Relation between the reaction time and learning effect. Reaction time in each block was normalized to satisfy the condition that the mean is zero and the standard deviation is one. (**E**) Number of incorrect trials for each participant during the baseline and probe trials when the white cursor was invisible. (**F**) Relation between the averaged movement angle in the pro- and anti-reaching movements across all training trials in each set. (**G**) Relation between the standard deviation of the movement angle in the pro- and anti-reaching movements during the training trials in each set.

When a prediction error *e*_*t*_ = *p*_*t*_ − *x*_*t*_ was observed (*p*_*t*_ indicated the degree of visuomotor rotation at the *t*th trial), ***W***_*t*_ was modified as

1 $${{\boldsymbol{W}}}_{t+1}={\rm{\lambda }}{{\boldsymbol{W}}}_{t}+\frac{\eta }{N}{e}_{t}{{\boldsymbol{A}}}_{t}^{T},$$

where λ and *η* indicate the retention and learning rate, respectively. In the current study, we focused on learning effects after learning processes converged (i.e., prediction error converged to certain value) with single target (Figs 2A,B and 6A,B). When learning processes converged with single target, ***W*** could be considered to converge to an equilibrium W^*^ with neural activities independent of trial number (i.e., ${{\boldsymbol{W}}}^{\ast }={\rm{\lambda }}{{\boldsymbol{W}}}^{\ast }+\frac{\eta }{N}e{{\boldsymbol{A}}}^{T}$). By solving the equation ${{\boldsymbol{W}}}^{\ast }={\rm{\lambda }}{{\boldsymbol{W}}}^{\ast }+\frac{\eta }{N}e{{\boldsymbol{A}}}^{T}$ with respect to W* and assuming a constant *p* that is independent of *t*, W* could be calculated as

2 $${{\boldsymbol{W}}}^{\ast }=\frac{\frac{\eta }{N}pE{[{\boldsymbol{A}}]}^{T}{(\mathrm{(1}-{\rm{\lambda }}){\boldsymbol{I}}+\frac{\eta }{N}{\rm{Cov}}({\boldsymbol{A}},{{\boldsymbol{A}}}^{T}))}^{-1}}{1+\frac{\eta }{N}E{[{\boldsymbol{A}}]}^{T}{(\mathrm{(1}-{\rm{\lambda }}){\boldsymbol{I}}+\frac{\eta }{N}{\rm{Cov}}({\boldsymbol{A}},{{\boldsymbol{A}}}^{T}))}^{-1}E[{\boldsymbol{A}}]},$$

where ***I*** is an identity matrix, *E*[***A***] denotes the ensemble average across all possible realizations and Cov(***A***, ***A***^*T*^) denotes the covariance between ***A*** and ***A***^*T*^. The *i*th component of *E*[***A***] was *E*[*A*_*i*_] = *A*_*i*_ + *E*[*ξ*_*i*,*t*_] = *A*_*i*_ and (*i*, *j*) th component of Cov(***A***, ***A***^*T*^) was Cov(*A*_*i*_, *A*_*j*_) = *E*[(*A*_*i*_ + *ξ*_*i*,*t*_)(*A*_*j*_ + *ξ*_*j*,*t*_)] − *E*[(*A*_*i*_ + *ξ*_*i*,*t*_)]*E*[(*A*_*j*_ + *ξ*_*j*,*t*_)] = *σ*^2^*δ*_*ij*_. Under the abovementioned noise conditions, the equilibrium W^*^ could be further calculated as

3 $${{\boldsymbol{W}}}^{\ast }=\frac{\frac{\eta }{N}pE{[{\boldsymbol{A}}]}^{T}}{1-{\rm{\lambda }}+\frac{\eta }{N}{\sigma }^{2}+\frac{\eta }{N}E{[{\boldsymbol{A}}]}^{T}E[{\boldsymbol{A}}]}.$$

In the following, *k* ∈ {pro, anti} and *l* ∈ {pro, anti} indicated the type of required reaching movements in the learning and probe trials, respectively. With these notations, ***W*** achieved the equilibrium ${{\boldsymbol{W}}}_{k}^{\ast }$ after the learning process converged with reaching movements with type *k*. With ${{\boldsymbol{W}}}_{k}^{\ast }$, learning effects in probe trials (with reaching movements with type *l*) could be calculated as ${x}_{{\rm{k}}\to l}^{\ast }={{\boldsymbol{W}}}_{k}^{\ast }{{\boldsymbol{A}}}_{l}$. Thus, the transfer of learning effects from the reaching movements with type *k* to those with type *l* could thus be written as follows:

4 $${x}_{{\rm{k}}\to {\rm{l}}}^{\ast }={{\boldsymbol{W}}}_{k}^{\ast }{{\boldsymbol{A}}}_{l}=\frac{\frac{\eta }{N}pE{[{{\boldsymbol{A}}}_{k}]}^{T}{{\boldsymbol{A}}}_{l}}{1-{\rm{\lambda }}+\frac{\eta }{N}{\sigma }^{2}+\frac{\eta }{N}E{[{{\boldsymbol{A}}}_{k}]}^{T}E[{{\boldsymbol{A}}}_{k}]}\mathrm{.}$$

The current study focused on whether the transfer was partial or perfect, which indicated that the primary focus is the ratio of the learning effects in the *k*th and *l*th type of reaching movements after training with the *k*th type of reaching movements, $\frac{{x}_{{\rm{k}}\to l}^{\ast }}{{x}_{{\rm{k}}\to k}^{\ast }}$. If this ratio was one, the transfer was perfect. Similarly, if the ratio was less than one, the transfer was partial. The ratio could be calculated as

5 $$\frac{{x}_{{\rm{k}}\to {\rm{l}}}^{\ast }}{{x}_{{\rm{k}}\to {\rm{k}}}^{\ast }}=\frac{E{[{{\boldsymbol{A}}}_{k}]}^{T}{{\boldsymbol{A}}}_{l}}{E{[{{\boldsymbol{A}}}_{k}]}^{T}E[{{\boldsymbol{A}}}_{k}]}=\frac{|{{\boldsymbol{A}}}_{l}|}{|E[{{\boldsymbol{A}}}_{k}]|}\,\cos \,\theta ,$$

where |***A***_*k*_| denotes the squared norm of ***A***_*k*_; $|{{\boldsymbol{A}}}_{k}|=\sqrt{{A}_{k1}^{2}+{A}_{k2}^{2}+\mathrm{...}+{A}_{kN}^{2}}$ and *θ* denote the angle between |*E*[***A***_*k*_]| and ***A***_*l*_.

#### A simple example of A

The current study focused on a simple model of neural activity *A*_*i*_ = *α*_*i*_ + *b* + *ξ*_*i*,*t*_, where *α*_*i*_ and *b* denote a local activity that was specific to the *i* th neural unit and a baseline that was common across all the neural units, respectively. In previous studies^4,6,7,20^, *A*_*i*_(*d*) was modeled as *m*_*i* _*f*_*i*_(*d* − *φ*_*i*_) + *b*, where *d*, *m*_*i*_, *f*_*i*_, *φ*_*i*_ and *b* are the desired movement direction (i.e., target direction), tuning height, tuning curve (e.g., a Gaussian function), preferred direction and baseline. The current study focused on a single planned movement direction *d*_0_, *A*_*i*_(*d*_0_) = *m*_*i* _*f*_*i*_(*d*_0_ − *φ*_*i*_) + *b* across all trials. Thus, we simplified this equation as *A*_*i*_(*θ*_0_) = *α*_*i*_ + *b* + *ξ*_*i*,*t*_, where *α*_*i*_ = *m*_*i* _*f*_*i*_(*θ*_0_ − *φ*_*i*_) (i.e., *α*_*i*_ represented a value that simultaneously consider tuning height, tuning width and preferred direction) and investigated several possible modulations of *α*, *b* and *ξ* in the pro- and anti-reaching movements.

For simplicity, we assumed $\frac{1}{N}{\sum }_{i=1}^{N}{\alpha }_{i}=0$, $\frac{1}{N}{\sum }_{i=1}^{N}{\alpha }_{i,k}{\alpha }_{i,l}=0$ and $\frac{1}{N}{\sum }_{i=1}^{N}{\xi }_{i,t}=0$ in the following calculations. The following calculations could be generalized without these simplifications.

#### A tuning-curve modulation model

In the tuning-curve modulation model, *α*_*i*_ differed between the pro- and anti-reaching movements, i.e., *α*_*i*,pro_ ≠ *α*_*i*,anti_, *b*_pro_ = *b*_anti_ = *b* and *ξ*_*i*,*t*,pro_ = *ξ*_*i*,*t*,anti_ = 0 (Fig. 5A). Thus, there was a balance in the population activity between the pro- and anti-reaching movements; $\frac{1}{N}{\sum }_{i=1}^{N}{A}_{{\rm{pro}},{\rm{i}}}=\frac{1}{N}{\sum }_{i=1}^{N}{A}_{{\rm{anti}},{\rm{i}}}$. In the simulation of experiment 1, the ratio of the learning effects in the pro- and anti-reaching movements was calculated as

6 $$\frac{{x}_{{\rm{pro}}\to {\rm{anti}}}^{\ast }}{{x}_{{\rm{pro}}\to {\rm{pro}}}^{\ast }}=\frac{{b}^{2}}{{\rm{Var}}({\alpha }_{{\rm{pro}}})+{b}^{2}} < 1,$$

where Var(*α*) denotes the variance of *α*_*k*_. In the simulation of experiment 4, the ratio of the learning effects in the anti- and pro-reaching movements was calculated as

7 $$\frac{{x}_{{\rm{anti}}\to {\rm{pro}}}^{\ast }}{{x}_{{\rm{anti}}\to {\rm{anti}}}^{\ast }}=\frac{{b}^{2}}{{\rm{Var}}({\alpha }_{{\rm{anti}}})+{b}^{2}} < 1.$$

#### A global modulation model

In the global modulation model, *b* differed between the pro- and anti-reaching movements, i.e., *α*_*i*,pro_ = *α*_*i*,anti_ = *α*, *b*_pro_ ≠ *b*_anti_ and *ξ*_*i*,*t*,pro_ = *ξ*_*i*,*t*,anti_ = 0 (Fig. 5B). In the simulation of experiment 1, the ratio of the learning effects in the pro- and anti-reaching movements was calculated as

8 $$\frac{{x}_{{\rm{pro}}\to {\rm{anti}}}^{\ast }}{{x}_{{\rm{pro}}\to {\rm{pro}}}^{\ast }}=\frac{{\rm{Var}}(\alpha )+{b}_{{\rm{pro}}}{b}_{{\rm{anti}}}}{{\rm{Var}}(\alpha )+{b}_{{\rm{pro}}}^{2}}.$$

In the simulation of experiment 4, the ratio of the learning effects in the anti- and pro-reaching movements was calculated as

9 $$\frac{{x}_{{\rm{anti}}\to {\rm{pro}}}^{\ast }}{{x}_{{\rm{anti}}\to {\rm{anti}}}^{\ast }}=\frac{{\rm{Var}}(\alpha )+{b}_{{\rm{pro}}}{b}_{{\rm{anti}}}}{{\rm{Var}}(\alpha )+{b}_{{\rm{anti}}}^{2}}\mathrm{.}$$

When $\frac{{x}_{{\rm{pro}}\to {\rm{anti}}}^{\ast }}{{x}_{{\rm{pro}}\to {\rm{pro}}}^{\ast }} < 1$ or *b*_p*ro*_ > *b*_anti_, $\frac{{x}_{{\rm{pro}}\to {\rm{anti}}}^{\ast }}{{x}_{{\rm{pro}}\to {\rm{pro}}}^{\ast }} > 1$.

#### An aiming-direction fluctuation model

In the aiming-direction fluctuation model, *ξ*_*i*,*t*,anti_ ≠ 0, the mean of *ξ*_*i*,*t*,anti_ was 0 and the variance of *ξ*_*i*,*t*,anti_ was *σ*^2^ (Fig. 5C). In the simulation of experiment 1, the ratio of the learning effects in the pro- and anti-reaching movements was calculated as

10 $$\frac{{x}_{{\rm{pro}}\to {\rm{anti}}}^{\ast }}{{x}_{{\rm{pro}}\to {\rm{pro}}}^{\ast }}=\frac{{\rm{Var}}(\alpha )+{b}^{2}}{{\rm{Var}}(\alpha )+{b}^{2}}=1,$$

indicating that this aiming-direction fluctuation model could not explain the results of experiment 1. In the simulation of experiment 4, the ratio of the learning effects in the anti- and pro-reaching movements was calculated as

11 $$\frac{{x}_{{\rm{anti}}\to {\rm{pro}}}^{\ast }}{{x}_{{\rm{anti}}\to {\rm{anti}}}^{\ast }}=\frac{{\rm{Var}}(\alpha )+{b}^{2}}{{\rm{Var}}(\alpha )+{b}^{2}}=1.$$

### Model parameters

As shown in Fig. 5, the transfer of the learning effects was simulated with *N* = 1000. The retention and learning rate, *λ* and *η*, were set to the best parameters that fit the learning curve, as shown in Figs 2A and 6A. In the tuning-curve modulation model, Var(*α*_pro_) = Var(*α*_anti_) and *b* were set to 0.055^2^ and 0.1, respectively. In the global modulation model, Var(*α*), *b*_pro_ and *b*_anti_ were set to 0.055^2^, 0.1 and 0.07, respectively. In the aiming-direction fluctuation model, Var(*α*), *b* and *σ*^2^ were set to 0.055^2^, 0.1 and 0.055^2^, respectively.

## Results

### Partial rather than perfect transfer of learning effects from pro- to anti-reaching movements

We evaluated the transfer of the learning effects from the pro- to anti-reaching movements in the same required planned movement direction using a visuomotor rotation of up to 15 degrees. Figure 2A shows the learning curves in the training trials when either the abruptly applied visuomotor rotation (blue) or gradually increasing visuomotor rotation (cyan) was imposed. Figure 2A also shows the learning effects in the probe trials in the pro- and anti-reaching movements toward a trained movement direction (green and red solid lines indicate the mean ± s.e.m., N = 10). To calculate the learning effects, we removed the incorrect trials (6 of 120 learning trials were determined to be incorrect). Figure 2B shows the averaged learning effects of each participant across the successful probe trials when either the abruptly applied visuomotor rotation (blue) or gradually increasing visuomotor rotation (cyan) was imposed in the training trials.

There was a significant interaction between ‘Type of reaching movements’ and ‘Movement direction’ (see *Statistical tests* for details, F(1,9) = 21.79, p = 0.00117). In particular, there was a significant difference in the learning effects between the pro- and anti-reaching movements toward the trained movement direction (N = 10, p = 6.70 × 10^−6^, Fig. 2C left and center left panels). The learning effects observed in pro-reaching movements were 12.5 ± 0.4 degrees (mean ± s.e.m.) and those in anti-reaching movements were 9.3 ± 0.4 degrees (mean ± s.e.m.). The learning effects trained with the pro-reaching movements were thus partially transferred to the anti-reaching movements in the same required planned movement direction. Figure 2C shows the averaged trajectories and learning effects in each participant and the movement trajectories also supported this partial transfer. This result indicated that rule switching might affect motor learning.

In addition to rule switching, several factors could explain the partial transfer as follows: (1) the difference in the visual cues between the pro- and anti-reaching tasks affected motor learning; (2) the cognitive demand affected motor learning, (3) the fluctuation in the planned movement direction in the anti-reaching movements affected motor learning and (4) the reaching movements toward the location without any visual cue (i.e., invisible target) in the anti-reaching tasks affected motor learning. We discuss these possibilities below.

To determine how the difference in the visual cues affects motor learning (e.g., position of the visual cue), we investigated the transfer of the learning effects from the pro-reaching movements toward a trained movement direction to anti-reaching movements toward an untrained movement direction and the transfer from the pro-reaching movements toward a trained movement direction to the pro-reaching movements toward an untrained movement direction. If the visual cue affected motor learning, the former could be expected to be larger than the latter because the visual cue was congruent in the pro-reaching movements toward the trained direction and anti-reaching movements toward the opposite direction. Our following results did not support this hypothesis. There was no significant difference between the two types of transfers (N = 10, p = 0.732, Fig. 2C center right and right panels).

To investigate the difference in the cognitive demand between the pro- and anti-reaching movements, we calculated the reaction time and the number of incorrect trials. The reaction time in the anti-reaching movements was significantly slower than that in the pro-reaching movements during the probe trials (two-sample t-test, N = 240 for pro- and N = 234 for anti-reaching movements, p = 1.14 × 10^−7^, means for pro- and anti-reaching movements were 190.0 and 206.0 ms, respectively), which is consistent with previous studies^10,11^. Due to a potential relation between the learning effects and the reaction time, we calculated the learning effects during the fast and slow reaction time trials in the pro- and anti-reaching movements. There was no significant difference in the averaged learning effects across the probe trials with the faster and slower reaction times (no interaction between ‘Type of reaching movements’ and ‘Reaction time’ [fast or slow] [F(1,9) = 0.171, p = 0.689] and the influence of ‘Reaction time’ was nonsignificant [F(1,9) = 0.210, p = 0.657]). Figure 2D shows the relation between the learning effects and the normalized reaction times with a mean of zero and standard deviation of one. There was no significant correlation between the reaction time and learning effects in reaching toward the trained movement direction (N = 240, r = −0.0160, p = 0.8050 for pro-reaching movements, N = 234, r = −0.0118, p = 0.858 for anti-reaching movements). Additionally, we investigated the number of incorrect trials in the pro- and anti-reaching movements during the baseline and probe trials when the cursor disappeared because this number might be indicative of certain aspects of cognitive demand. There was no significant difference in the number of incorrect trials between the pro- and anti-reaching movements (Fig. 2E, F(1,9) = 1.8299, p = 0.209). In experiment 1, no incorrect trial was detected in the probe trials with pro-reaching movements, indicating that some incorrect trials with pro-reaching movements occurred during the baseline trials. Although the reaction time might suggest more cognitive demand in the anti- than that in the pro-reaching movements, these results indicated that the cognitive demand might not be significantly relevant in the learning effects in our experimental setting.

Figure 2F demonstrates the difference between the target location and the averaged movement direction of each participant across the baseline trials between the pro- and anti-reaching movements. These values indicate the difference between the actual and required movement directions. There was no significant difference in the movement directions between the pro- and anti-reaching movements during the baseline trials (F(1,9) = 0.721, p = 0.418), which indicated that the fluctuation in the planned movement direction in the anti-reaching movements was not significantly different from that in the pro-reaching movements. Figure 2G demonstrates the standard deviation of the movement angle in the pro- and anti-reaching movements. There was no significant difference in the standard deviations (F(1,9) = 0.159, p = 0.699). These results indicated that the fluctuation of the planned movement direction was not highly relevant to the partial transfer in the current experiment.

**Figure 3:**
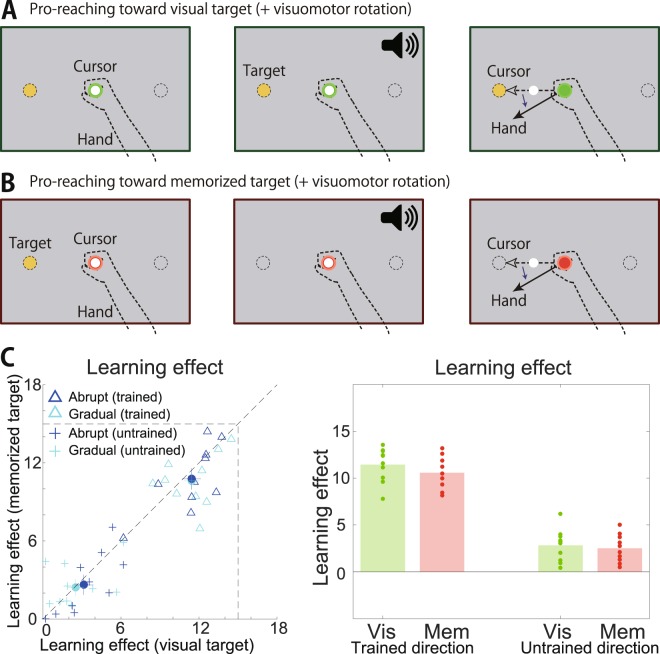
Transfer of learning effects from the reaching movements toward the visible target to those toward the invisible and to-be-memorized targets in experiment 2. (**A**) When the color of the center circle was green, the participants were required to perform arm-reaching movements to move the white cursor toward the yellow visual cue. (**B**) When the color of the center circle was red, the yellow visual cue was visible for 400 ms and subsequently disappeared. Participants were required to memorize the location of the cue and perform arm-reaching movements to move the white cursor toward the memorized cue location after a beep sounded. The beep sounded 600 ms after the disappearance of the cue. (**C**) Learning effects in reaching toward visual and to-be-memorized target cues.

We conducted experiment 2 to investigate how reaching movements toward an invisible target impacted the learning effects. In the pro-reaching movements, the participants were required to reach for visible targets. In contrast, in the anti-reaching movements, the participants were required to reach for invisible targets in the current experimental setting. In addition to the switching rule, the differences between the reaching movements toward visible and invisible targets could affect the transfer of the learning effects. In experiment 2, the participants reached toward either a visible target or invisible and to-be-memorized targets (Fig. 3A,B). Participants adapted to a visuomotor rotation in the reaching movements toward a visible target and the transfer of the learning effects from the reaching movement toward the visible target to those toward the invisible and to-be-memorized targets was investigated. In contrast to the results of experiment 1, the transfer was perfect rather than partial (Fig. 3C, there was no significant interaction [all the F(1,9) were smaller than 0.585] and p = 0.217 for the influence of ‘Type of reaching movements’ on learning effects in trained movement directions). Thus, we concluded that the reaching movements toward an invisible target were not a significant factor in the partial transfer from the pro- to anti-reaching movements.

### Switching rules caused the partial availability of motor learning

**Figure 4:**
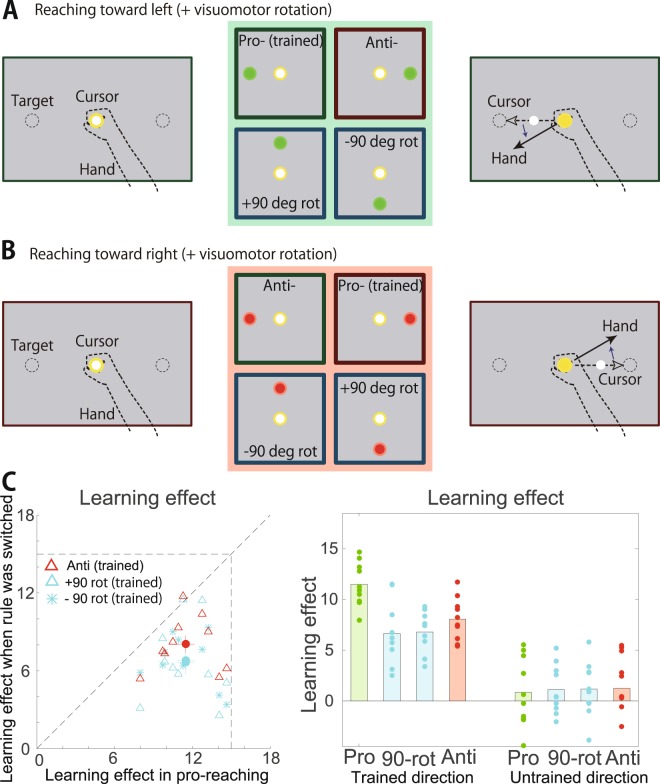
Transfer of learning effects when the rules were switched in experiment 3. (**A**) When the color of the visual cue was green, the participants were required to perform reaching movements to move the white cursor toward the 180° direction. (**B**) When the color of the visual cue was red, the participants were required to perform reaching movements to move the white cursor toward the 0° direction. (**C**) Learning effects in the pro-reaching movements, reaching movements with ±90° rotation and anti-reaching movements.

To investigate whether switching rules affects motor learning again and whether anti-reaching is a specific factor that affects motor learning, we conducted experiment 3 (Fig. 4). In this experiment, the participants were required to reach for the 0° location when the target color was green independently of the location of the visual cue. When the color was red, the participants were required to reach for the 180° location independently of the location of the visual cue. This experimental setting included not only anti-reaching but also other types of rules (i.e., the color of the visual cue was a determinant of the required movement direction) (Fig. 4A,B). Participants adapted to the visuomotor rotation with pro-reaching movements. Then, we investigated the transfer of the learning effects from the pro-reaching movement to other types of reaching movements. There was a significant interaction between ‘Type of reaching movements’ and ‘Movement direction’ (see *Statistical tests*, F(1,9) = 7.6691, p = 7.31 × 10^−4^). The learning effects obtained with the pro-reaching movements were partially rather than perfectly transferred to other types of reaching movements involving switching rules (Fig. 4C, N = 10, p = 0.0127 for reaching movements with a 90 degree rotation, N = 10, p = 0.00711 for reaching movements with a −90 degree rotation and N = 10, p = 0.0201 for anti-reaching movements). There was no significant difference between the learning effects in the reaching movements with a 90 degree rotation and those in the anti-reaching movements (N = 10, p = 0.106), between the reaching movements with a −90 degree rotation and those in the anti-reaching movements (N = 10, p = 0.226) and between those with a 90 degree rotation and −90 degree rotation (N = 10, p = 0.999). The partial transfer from the pro-reaching movement to other types of reaching indicated that anti-reaching was not a specific factor that induces a partial transfer. Therefore, the switching rules affected motor learning and caused the partial availability of learning effects.

### Several computational models to explain the partial transfer from pro- to anti-reaching movements and to predict the transfer from anti- to pro-reaching movements

To further investigate the effects of rule switching, we proposed a computational framework to explain the partial transfer of the learning effects from the pro- to anti-reaching movements. Several computational models were considered to explain the partial transfer from the pro- to anti-reaching movements as follows: (1) the tuning-curve modulation model in which the movement-direction-dependent components in neural activities were different between the pro- and anti-reaching movements, (2) the global modulation model in which the neural activities were larger in the pro-reaching than those in the anti-reaching movements and (3) the aiming-direction fluctuation model in which the planned movement direction showed trial-to-trial variations in the anti-reaching movements.

We investigated several computational models that relied on the motor primitive framework in which a linear combination of neural activities generates motor commands and the linear weightings were updated to minimize the prediction error^4,6,7,20^, which corresponds to the state-space model that is frequently used in modeling motor learning^2,20^. We assumed that the activity of the *i* th motor primitive in pro- and anti-reaching movements, *A*_pro,i_ and *A*_anti,i_, were modeled as *A*_pro,i_ = *α*_pro,i_ + *b*_pro_ + *ξ*_pro,i,*t*_ and *A*_anti,i_ = *α*_anti,i_ + *b*_anti_ + *ξ*_anti,i,*t*_, respectively, where *α* indicated the tuning height, which was different for each neural unit; *b* indicated the baseline activity, which was the same across all neural units; and *ξ* indicated the trial-to-trial variant noise.

**Figure 5:**
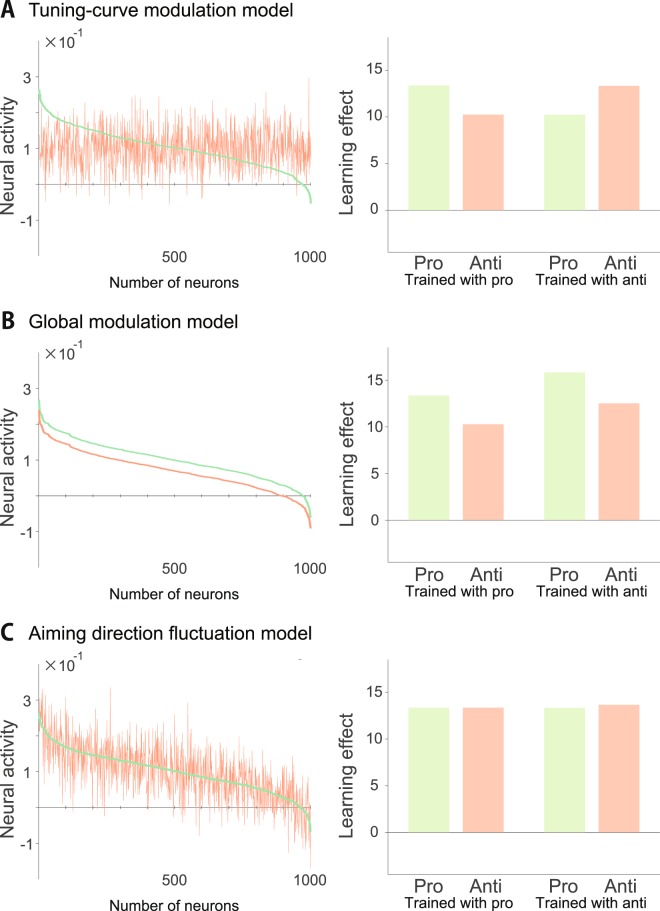
Simulations of several computational models. (**A**) Tuning-curve modulation model. (Left): Neural activities in simulated pro- (green) and anti-reaching movements (red). Horizontal and vertical axes denote neuron numbers and neural activities, respectively. (Right): Simulated learning effects in pro- and anti-reaching movements when trained with either pro- or anti-reaching movements. (**B**) Global modulation model. (**C**) Aiming-direction fluctuation model.

In the tuning-curve modulation model, where *α*_pro,i_ ≠ *α*_anti,i_, *b*_pro_ = *b*_anti_ and *ξ*_pro,i,*t*_ = *ξ*_anti,i,*t*_ = 0 (Fig. 5A), the transfer of learning effects from the pro- to anti-reaching movements was partial (Fig. 5A, Eq. 6). The tuning-curve modulation model yielded a prediction regarding the transfer from the anti- to pro-reaching movements, wherein the transfer could be partial (Fig. 5A, Eq. 7).

In the global modulation model, where *α*_pro,i_ = *α*_anti,i_, *β*_pro_ > *β*_anti_ and *ξ*_pro,i,*t*_ = *ξ*_anti,i,*t*_ = 0 (Fig. 5B), the transfer of learning effects from pro- to anti-reaching movements was partial (Fig. 5B, Eq. 8). The global modulation model yielded a prediction about the transfer from the anti- to pro-reaching movements, wherein the transfer could be over 100% (Fig. 5B, Eq. 9).

In the aiming-direction fluctuation model, where *α*_pro,i_ = *α*_anti,i_, *b*_pro_ = *b*_anti_, *ξ*_pro,i,*t*_ = 0 and *ξ*_anti,i,*t*_ ≠ 0 (Fig. 5C), the transfer of learning effects from pro- to anti-reaching movements was perfect (Fig. 5C, Eq. 10). Because this result was inconcistent with our results, the fluctuation of the aiming direction was not considered a significant factor explaining the partial transfer from the pro- to anti-reaching movements. The aiming-direction fluctuation model yielded a prediction about the transfer from the anti- to pro-reaching movements, wherein the transfer could be perfect (Fig. 5C, Eq. 11).

### Validation of the tuning-curve modulation model

**Figure 6:**
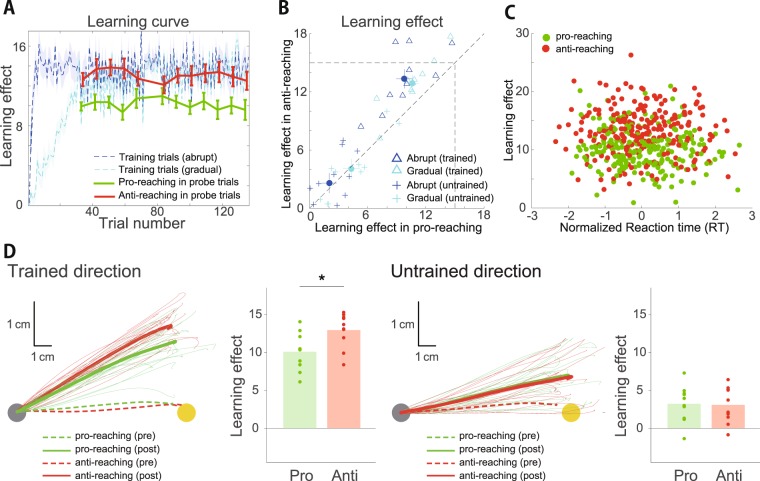
Transfer of learning effects from anti- to pro-reaching movements. (**A**) Learning curves during the training and probe trials of the anti-reaching movements. (**B**) Learning effects in each subject. (**C**) Relation between the reaction time and learning effect. Reaction time in each block was normalized to satisfy the condition that the mean is zero and the standard deviation is 1. (**D**) Trajectories of reaching movements and learning effects. All trajectories were arranged to be directed toward an up-rightward direction.

Because three computational models generated different predictions about the transfer from anti- to pro-reaching movements, we conducted experiment 4 to investigate which prediction was valid. In experiment 4, the participants adapted to the visuomotor rotation in anti-reaching movements. Then, we investigated the transfer from anti- to pro-reaching movements. There was a significant interaction between ‘Type of reaching movements’ and ‘Movement direction’ (see *Statistical tests* for details, F(1,9) = 12.2, p = 0.00674). In contrast to the results of experiment 1, there was a significant interaction between ‘Movement direction’ and ‘Type of perturbation’ (see *Statistical tests* for details, F(1,9) = 12.7, p = 0.00603). Our results indicated that the transfer from anti- to pro-reaching movements was partial rather than perfect (Fig. 6, N = 10, p = 9.95 × 10^−5^ for reaching movements toward the trained movement direction). Among the models of tuning-curve modulation, global modulation and aiming-direction fluctuation, only the tuning-curve modulation model satisfied this condition. The tuning-curve modulation model indicates not only the modulation of each neural activity but also the balance of the population activity between the pro- and anti-reaching movements; *A*_pro,i_ can be different from *A*_anti,i_ while satisfying $\frac{1}{N}{\sum }_{i=1}^{N}{A}_{{\rm{pro}},{\rm{i}}}=\frac{1}{N}{\sum }_{i\mathrm{=1}}^{N}{A}_{{\rm{anti}},{\rm{i}}}$. Additionally, there was no significant difference between the degree of transfer from the pro- to anti-reaching (0.7618 ± 0.0338 [mean ± s.e.m.]) and from the anti- to pro-reaching movements (0.7826 ± 0.023 [mean ± s.e.m.]) (N = 10, p = 0.635, two-way ANOVA followed by Tukey’s post hoc comparison, no interaction between ‘Experiment number’ (1 or 4) and ‘Type of perturbation’ (F(1,36) = 1.11, p = 0.299)). Thus, the tuning-curve modulation model predicted that neural activities in the pro- and anti-reaching movements would overlap by approximately 75−80%.

In experiment 4, the participants experienced more anti-reaching movement trials than pro-reaching movement trials. The reaction time could be expected to be the same or faster in the anti-reaching movements than that in the pro-reaching movements. However, the reaction time remained slower in the anti-reaching movements than that in the pro-reaching movements during the probe trials (two-sample t-test, N = 232 and N = 238 for pro- and anti-reaching, respectively, p = 4.1685 × 10^−4^, means for pro- and anti-reaching movements were 193.6 ms and 206.8 ms, respectively), indicating that the cognitive demand might be larger in the anti-reaching movements. If cognitive demand affected motor learning, the transfer from anti- to pro-reaching movements should be larger than 100% or close to 100%. Because we could not find this tendency, cognitive demand was not considered a significant factor affecting motor learning in switching rules in the current experimental setting.

Additionally, there was no significant difference in the number of incorrect trials (F(1,9) = 1.23, p = 0.297), there was no significant difference in the movement directions between the pro- and anti-reaching movements during the baseline trials (F(1,9) = 1.96, p = 0.196), or there was no significant difference in the standard deviations of the movement angle between the two types of reaching movements (F(1,9) = 0.00734, p = 0.934).

## Discussion

The current study investigated the influence of switching rules on motor learning in the same required planned movement and we observed that switching rules decreased the learning effects. A decrease in learning effects was observed during the transfer of learning effects from pro- to anti-reaching movements (Fig. 2), from pro-reaching to reaching movements under several types of rules (Fig. 4) and from anti- to pro-reaching movements (Fig. 6) was investigated. The decrease in learning effects might not be attributed to the reaching movements toward the location without any visual cue (Fig. 3), cognitive demand (Figs 2D,E and 6C) and fluctuation of aiming direction (Fig. 2F,G). We additionally proposed a computational model to explain the influence of switching rules on motor learning. The tuning-curve modulation model explained and predicted our experimental results. In this model, each neural unit shows different neural activities, but the neural population demonstrated the same mean activity between the pro- and anti-reaching movements; *A*_pro,i_ ≠ *A*_anti,i_ and $\frac{1}{N}{\sum }_{i=1}^{N}{A}_{{\rm{pro}},{\rm{i}}}=\frac{1}{N}{\sum }_{i=1}^{N}{A}_{{\rm{anti}},{\rm{i}}}$. Our results suggested that neural units show different tuning curves for the same planned movements but different rules; however, the mean activities in the population have similar values.

### Partial availability of learning effects in the same planned movements with different rules

A principal factor in determining the availability of motor learning effects is the planned movement direction^6–9^. After the training, the learning effects are partially available in the probe trials when the movement directions are different between the trained and probed movement directions^6,7^. Even when the executed movement directions are the same, the participants can learn different motor tasks when the planned movement directions are different^8,9^. Based on these results, we expected the same amount of motor learning effects in the pro- and anti-reaching movements when the same movement directions were required. However, the current study revealed that the availability of motor learning effects was partial rather than perfect when the same planned movement directions were needed, but the rule was switched (Figs 2, 4 and 6). Our results indicate that the partial availability originated from the modulation of neural activities when the same planned movement directions were required, but the rule was switched. The modulation was observed in the pro- and anti-saccade, pro- and anti-reaching and several brain regions related to saccade or arm-reaching movements^10,11,15,16,21^.

### Relation to the influence of context on motor learning

The switching rule can be a context and its influence on motor learning was investigated in several previous studies^8,9,13,14,22–25^. In saccades, especially in reactive but not spontaneous saccades, the learning effects obtained in one context were perfectly rather than partially available in another context when required saccades were the same among the contexts. Participants could switch the learning memory depending on the context or target stimulus, such as a flickering stimulus or steady stimulus, even when the same saccades were required in both stimuli^22^. Although participants could switch the memory, the transfer of learning effects obtained in one context was perfectly available in the other context. Previous studies investigated the transfer of learning effects from pro- to anti-saccade using saccadic gain adaptation^13,14^, which suggested the perfect transfer from pro- to anti-saccades. In sum, those previous studies have reported the perfect transfer of learning effects among the context (i.e., stimulus type or rule type) when the required motion was the same. Conversely, the current study reported a partial transfer of learning effects even when the required motion was the same. Although the partial transfer (i.e., partial switch of motor memory) among different contexts were reported in saccades^23^ and arm-reaching movements^8,24,25^, the partial transfer was among the context with different required motions, different initial hand positions, or different movement effectors (e.g., unimanual or bimanual arm-reaching movements). The current study reported a partial transfer under the same required motion, the same initial hand position and the same movement effector. Because the perfect transfer could be observed in experiment 2 (reaching movements toward either visual target or invisible target), the partial transfer under the same required motion, the same initial hand position and the same movement effector could be induced by the switching rule, especially in arm-reaching movements.

### The tuning-curve modulation model but not the global model explained and predicted the current results

The current results suggested that the mean activities of the neural population were similar between the pro- and anti-reaching movements; $\frac{1}{N}{\sum }_{i=1}^{N}{A}_{{\rm{pro}},{\rm{i}}}=\frac{1}{N}{\sum }_{i=1}^{N}{A}_{{\rm{anti}},{\rm{i}}}$, which was also suggested by our previous models for motor learning in unimanual and bimanual movements^26,27^. Pro- and anti-saccade movement-related neural activities have been widely investigated. A previous study reported that activities in the superior colliculus and frontal eye field were smaller in anti-saccade movements than those in pro-saccade movements^11^. Another study reported that neural activities in the supplemental eye field were higher in anti-saccade movements than those in pro-saccade movements^21^. Based on these findings related to saccades, it could be predicted that neural units exhibited a lower or higher activities in the anti-reaching movements than in the pro-reaching movements. The current study supported the hypothesis that neural units exhibited higher or lower activities in the anti-reaching than in the pro-reaching movements, but the mean activities of the neural population were balanced between the two types of reaching movements. According to previous studies, each neural unit shows different neural activities between pro- and anti- arm-reaching movements^15,16^. The results of our experiments and computational models suggested that the summed neural activities are similar between the two types of reaching movements.

### Influence of cognitive demand on motor learning

Previous studies have reported that divided attention affects motor learning^28^; more required attention correlates with less motor adaptation. Anti-reaching movements might require higher attention levels than pro-reaching movements because the reaction time was significantly slower in the anti-reaching movements. If attention or cognitive demand affected motor learning in the current study, the learning effects would be larger in the trials with either faster or slower reaction times, the transfer from the anti- to the pro-reaching movements would be an over-generalization (a transfer rate larger than one), or the number of incorrect trials would be larger in the anti-reaching movements than that in the pro-reaching movements. Although there may be a difference in the attention requirement between the pro- and anti-reaching movements, we could not find a significant effect of the difference in the current study (Fig. 2). These results and our computational model indicate that the influence of the modulation of neural activities on motor learning likely overrides that of divided attention.

### Influence of explicit and implicit learning

Learning effects consist of (at least) two factors; explicit (cognitive) and implicit learning effects^3,17,18^. In a visuomotor rotation, participants can compensate for task errors between the target position and actual movement by aiming at a different location from the target position. The cognitive factor used to change the aiming direction is referred to as explicit learning, which is distinct from implicit learning in which movement is changed to minimize prediction errors without awareness. In our experiments, there was a possibility that subjects relied on explicit learning in the trained condition, while these explicit learning effects were unavailable in the probe trials, which would imply that the availability of learning effects in probe trials was partial rather than perfect. Possible arguments against this possibility are as follows; (1) instruction to strictly aim at the target, (2) visuomotor rotation of 15 degrees, (3), short preparation time and (4) unawareness of the perturbation. First, we instructed the participants to strictly aim at the target (visual cue in pro-reaching movements and the position opposite the cue in anti-reaching movements), consistent with the procedures used to investigate implicit learning components in previous studies^3,17^. Second, we used the visuomotor rotation of 15 degrees because explicit learning effects for adapting to a visuomotor rotation of 15 degrees were likely less than those for a visuomotor rotation of 30 degrees (Fig. 3 in^17^). Third, the movement preparation time was quite short in our experimental setting, as participants needed to move the cursor toward the target immediately after it appeared without any delay. Because a short preparation time can suppress explicit learning^18^, our results mainly reflect implicit learning effects. Fourth, no participant was aware of the visuomotor rotation even when the visuomotor rotation of 15 degrees was abruptly applied (N = 40). After each experiment, we asked the participants “Please tell us what you were aware of in this experiment”. No participants reported the existence of the visuomotor rotation. Because unaware perturbation cannot be explicitly compensated for, our results mainly reflect implicit learning effects.

### Relation between arm-reaching movements and saccades

A previous study investigated the transfer of learning effects from pro- to anti-saccades using saccadic gain adaptation^13,14^. The difference between saccadic gain in pre-learning and that in post-learning was the same as that between pro- and anti-saccades, which suggested the perfect transfer from pro- to anti-saccades. Although this result may be inconsistent with the current results, several factors could potentially explain this difference. First, the difference in the effector for the required movements may be an explanatory factor. The previous study relied on saccades and the current study relied on arm-reaching movements. This possibility can be considered because neurons in posterior parietal cortex and parietal reaching region have different activities for saccade and arm-reaching movements^29,30^. Furthermore, the difference in the adaptation task may be an explanatory factor. The previous study relied on gain adaptation or an adaptation of the movement distance. In contrast, the current study focused on adaptation to the visuomotor rotation because several previous models have focused on adaptation to the angular deviation or deviation perpendicular to the movement direction^4–7^. Several previous studies have reported that the transfer of learning effects from a trained movement to an untrained (probed) movement was different between gain adaptation and the adaptation to the visuomotor rotation^12^, indicating that the neural mechanisms underlying these adaptations are different. The difference in both the effector and adaptation task could contribute to the difference between the results of the previous studies^13,14^ and the current results.

## Electronic supplementary material

Program code

## Data Availability

All data generated or analyzed during this study are included in this article.
